# Hospital Pharmacists and Food Economy

**Published:** 1917-05-12

**Authors:** 


### HOSPITAL PHARMACISTS AND FOOD ECONOMY.

Economy has always been the keynote of work in the dispensaries of our hospitals. Pharmacists, by the use of substitutes for certain non-essentials, such as alcohol in various preparations, the use of liquid extracts instead of the corresponding tinctures, and essential oils to replace the spirituous solutions, have saved their institutions much money, without detriment to the therapeutic effect of the prescribed articles.

Now, however, in view of the growing shortage of food it becomes necessary to eliminate all preparations which can be used as foods and are not really essential to medical treatment.

We take as an example lard. This is used either plain or benzoated in huge quantities in the form of zinc ointment and similar preparations for external application, and there appears to be no valid reason why *all* this lard should not be saved for human consumption by the substitution of a soft paraffin base, more familiar as the proprietary "Vaseline."

It may be argued that this base, not being "absorbed," is unsuitable, but it does not seem essential that oxide of zinc, at any rate, should have an "absorbent" base. We estimate that at a fair-sized hospital about 56 lb. of lard would be set free every week for food purposes if the procedure suggested were adopted. Another article used largely is a dusting powder of oxide of zinc and starch. It would appear that the new Order made by the Food Controller should be here applied, and no starch used for this purpose in future.

Hospital pharmacists can easily devise a suitable substitute—say talc, which is used largely in America for dusting purposes. It makes a good powder for general use, and has the advantage of being quite cheap.

Again, now that glycerine is practically unobtainable, greater quantities of olive, almond, nut, and other vegetable oils are being used, but it should be noted that these substances are all adaptable for food purposes, and therefore should only be put to medical and surgical use.

We *have* heard of olive oil being used for polishing ward tables, etc., and would suggest that any such use is now, if not before the war, extravagant, and in direct opposition to the wishes of the Food Controller.

These few suggestions are put forward in the hope that our pharmacist readers, more especially, may be stimulated to go into the matter and consider whether they can aid the work of those responsible for the conservation of our food supplies.

